# Comparative Analysis of Hydration Status and Microbial Quality of Tap Water Between Urban and Rural Settings in the Ashanti Region of Ghana

**DOI:** 10.1155/ijfo/4773110

**Published:** 2025-01-10

**Authors:** Marina A. Tandoh, Priscilla Owusu, Claire-Rush N. A. Nkrumah, Veronica Tawiah Annaful, Chris Y. Asare, Samuel Selorm Attu

**Affiliations:** ^1^Department of Biochemistry and Biotechnology, Kwame Nkrumah University of Science and Technology, Kumasi, Ghana; ^2^Department of Food Science, University of Arkansas, Fayetteville, Arkansas, USA

**Keywords:** communities, Ghana, hydration status, microbial quality, tap water

## Abstract

Maintaining proper hydration is crucial for human health, physiological functions, and cognitive performance. This study sought to determine the hydration status and the microbial safety of tap water in an urban setting (Kwame Nkrumah University of Science and Technology Campus) and a rural community (Adjamesu) in the Ashanti Region of Ghana. The water safety perception and hydration status of 171 study participants aged 10–61 years were assessed. Six taps were randomly selected at each site in triplicates, resulting in 36 water samples. The microbial quality of the water was assessed by testing for heterotrophic bacteria, coliforms, *Staphylococcus aureus*, and *Salmonella*. The physicochemical quality of the water samples was assessed with a pH meter. About 68.9%, 20%, and 11.1% of participants were minimally dehydrated, significantly dehydrated, and well hydrated, respectively. Furthermore, 86.7% of the urban participants did not depend on tap water (*p* < 0.001) compared to 46.9% of the rural participants (*p* = 0.579). Moreover, 78.9% of the urban participants did not trust the quality of tap water (*p* < 0.001), compared to 38.3% of rural dwellers (*p* = 0.035). The water microbial quality assessment of both the rural and urban water samples indicated the presence of some heterotrophic bacteria at similar levels (*p* = 0.0649) between 7.0 × 10^0^ ± 1.15 and 5.93 × 10^3^ ± 5.51 cfu/mL. Some coliforms in the samples exceeded safe limits with those of the rural communities recording higher levels of contamination. Further assessment revealed the absence of pathogenic bacteria such as *Escherichia coli*, *Salmonella*, or *Staphylococcus aureus*. However, regular monitoring and evaluation of tap water quality are crucial, given its significance as a primary hydration source for the general population. Additionally, it would be advisable for consumers to treat their water further when using it for cooking and drinking to prevent any adverse health effects.

## 1. Introduction

Water is vital to sustain life and maintain the growth and well-being of humans [1]. Water has numerous functions and is used in almost all human activities including cooking, washing, and bathing [2]. Globally, more than a billion people do not have access to a good water supply and more than two times that number lack basic sanitation [3]. Water is said to be a basic human right that is required for good health and well-being [4]. As a result, the United Nations General Assembly has put across measures to ensure that everyone has access to safe, clean, accessible, and cheap drinking water [5]. Groundwater, surface water, and rainwater are the most common drinking water sources, yet the quality of these sources varies [6]. Groundwater can easily be polluted with micro-organisms such as bacteria, viruses, and protozoa and chemical contaminants like pesticides and herbicides [6]. Sources of these contaminants are notably from dumpsite leakage and flow from leaking sanitation systems. Surface water undergoes regular treatment at water treatment plants before being distributed to consumers. However, the presence of established biofilms within water distribution lines leads to contamination during transmission, occurring even prior to reaching taps in homes and households [7]. This contamination poses significant health risks, as it can harbor infectious diseases like diarrhea, cholera, hepatitis A, dysentery, polio, and typhoid when consumed or utilized. Individuals who are exposed to unsafe water have a risk of encountering several diseases and in worse cases suffer death [1].

In Ghana, the main sources of water for both drinking and domestic use are pipe-borne water, wells, and natural sources such as rainwater and rivers/streams [8]. Other alternatives include bottled water, sachet water, and tanker water. In the Kumasi Metropolis, domestic and drinking water are obtained from self-water supply or water service delivery companies [9]. The most widely used water source is pipe-borne water, which serves various household needs. Mechanized and nonmechanized wells are also used for drinking by 12.6% of households while 14.7% use theirs for several domestic functions [10].

The hydration status of an individual is very important because lack of enough water potentially contributes to various diseases [11] and is linked to reduced physical activity and intellectual performance [12]. Dehydration results when there is an insufficient water intake relative to the volume of the bodily losses, and this is a significant health concern [13]. An individual's water intake is estimated to contribute approximately 20% of water from solid foods and 80% of water from drinking water and other liquid foods [14]. The hydration status of an individual is therefore dependent on their drinking and eating habits, choice of foods, and the availability and access to foods, water, and beverages [11]. The commonly used technique to measure changes in the hydration status of an individual is the measurement of body weight changes over periods [15]. There are other techniques for measuring the hydration status of an individual such as tracer techniques, urine indices, bioelectrical impedance, and lastly plasma or serum molarity (plasma indices) [15].

The quality of water has been reported to diminish from the moment it is collected to when it is used [16] due to storage and handling practices [17]. Hence, most people perceive that tap water is unsafe for drinking, and it deters many from using tap water as a drinking source. The goal of this study was to compare water intakes and the microbial safety of tap water between a typical rural community (Adjamesu) and an urban community (Kwame Nkrumah University of Science and Technology (KNUST) campus), both in the Kumasi Metropolis. Access to safe water must be of much importance to every individual because water is needed by the body daily to assist in important biological functions. The vital nature of water in metabolic activities requires that it is clean and safe to use [18]. Drinking unsafe water is associated with many diseases, especially stomach-related illnesses [18]. Good hydration among students is essential for optimal health and cognitive functioning [19]. Much work has been done with regard to the quality of water in societies but not so much has been done with regard to linking it with the hydration status of individuals. For instance, the safety of borehole water as an alternative source of drinking water in Kumasi assessed by Boadi et al. [20] revealed that 85% of the 20 samples were not safe for drinking due to their contamination with total and fecal coliforms. Aside microbial contamination, recent studies also reported contamination of water in the Kumasi Metropolis with heavy metals [21, 22] and other dissolved solids, making them unsuitable for drinking [22]. This work provides scientific data on the safety of tap water and the hydration status of individuals in some parts of the Kumasi Metropolis in the Ashanti Region of Ghana.

## 2. Materials and Methods

### 2.1. Study Design, Study Site, and Study Population

A cross-sectional study design was adopted to explore the hydration status and microbial quality of water for residents on KNUST campus and the Adjamesu community, off the Bekwai road, both located in the Ashanti Region of Ghana from June 2022 to September 2022.

### 2.2. Sample Size Calculation

Minimum sample size was obtained based on the sample size calculation using Cochran's formula [23]: *n* = *z*^2^*pq*/*e*^2^, where *n* is the sample size, *z* is the confidence level 95% (1.96), *p* is the expected proportion in population based on previous studies 90%, *q* is the predefined (1 − *p*), and *e* is the margin of error at 5%.

The prevalence of voluntary dehydration among a sample of Cypriot adolescents in a school was 90%:

$$\begin{array}{cc} \; & n=\left\{{\left(1.96\right)}^{2}*0.90*\left(1-0.90\right)\right\}/{\left(0.05\right)}^{2}\\ n=138.29\left(\text{approximately }138\text{ participants}\right). \end{array}$$

However, a total sample size of 90 was obtained for the urban community and 81 for the rural community in this study.

### 2.3. Inclusion and Exclusion Criteria

Any one of the taps in each of the six traditional halls on the KNUST campus was sampled. In the Adjamesu community, taps were randomly sampled. Taps with visible leakages were not sampled because leaks could introduce external contaminants to the water and alter water quality, thereby influencing results. Taps with attachment repairs owing to splashing were also not sampled as splashing could also introduce environmental contaminants and compromise the consistency of the sample collection and potentially increase microbial load which would not be reflective of the quality of the original water.

### 2.4. Data/Sample Collection and Analysis

A questionnaire was administered to all participants to assess their sources of drinking water, how they perceived the tap water, how well they consumed water, and their knowledge of hydration. Water samples were collected using sterile 1.5-L bottles for tap water testing. A total of 36 water samples were collected from six randomly selected taps in both rural and urban communities. One milliliter of water was collected from each tap in triplicates after being made to flow for 2–3 min before sampling, ensuring representativeness. Taps with leakages were excluded. Rigorous sterilization and cleaning of the tap nozzles using 70% ethanol were followed before water collection. The study design was a two-strata sample site (rural and urban), in which six taps were randomly selected at each site in triplicates resulting in a 2 × 6 × 3 factorial design using 36 samples.

The microbial quality assessment of the water samples included total heterotrophic bacteria count on plate count agar (PCA), total fecal coliform count on MacConkey agar (MA), *Staphylococcus aureus* on mannitol salt agar (MSA), and *Salmonella* on xylose lysine deoxycholate agar (XLD) (Oxoid Ltd, Basingstoke, United Kingdom). The water samples were diluted threefold in sterile 0.1 peptone water and aliquots of 1 mL of full strength and dilutions were plated on PCA, MSA, and MA using the spread plate technique. The *Salmonella* samples were pre-enriched in 1% peptone (Oxoid Ltd, Basingstoke, United Kingdom) for 24 h and further enriched in Rappaport Vassiliadis broth (Oxoid Ltd, Basingstoke, United Kingdom) for another 24 h subsequent to plating on XLD using 0.01-mL aliquots. Inoculated plates for PCA, MA, XLD, and MSA were incubated at 37°C for 24 and 48 h of observation. MA plates for fecal coliforms were incubated at 42°C ± 2 for 24 h [24] Plates were observed for colonies postincubation and enumerated while colony morphological traits on selective media were used in identification. Pure colonies of interest were subcultured unto nutrient agar (Oxoid Ltd, Basingstoke, United Kingdom) for Gram staining and biochemical tests (catalase and oxidase) as described by Bishankha et al. [25].

Participants provided “early morning” urine samples in sterile containers labeled with corresponding sample codes. Clear instructions were given for the collection process to avoid external contamination. The hydration status of the individuals was determined by comparing the specific gravity of the urine to the indexes of hydration status which classifies individuals' hydration status based on the specific gravity values as either “well hydrated,” “minimal dehydration,” or “significant dehydration or serious dehydration” [26]. The specific gravity of urine was determined using two methods: urinalysis test strips (URIT Medical Electronics Co. Ltd 24020036M1), which measured the unit of interest semiqualitatively, and a manual optical refractometer which measured quantitatively. Both methods were employed for each sample, providing reliable specific gravity measurements. pH levels of all water samples were determined using a pH meter (Mettler Toledo GR/T 11165) and indicator strips.

### 2.5. Statistical Analysis

Data was entered into Microsoft Excel 2016 and analyzed with the Statistical Package for the Social Sciences (SPSS) Version 24. GraphPad Prism 5.0 was used for processing the data obtained from the microbial load assessment at a 95% confidence interval using one-way analysis of variance (ANOVA). All analysis was conducted in triplicates, and the means were used in the analysis. Some analysis was also conducted using a two-way ANOVA tool of GraphPad Prism 5.0 at a 95% confidence interval with a significant difference established at *p* values less than 0.05. Fisher's exact test was used to compare the output of the urinalysis strip and refractometer.

### 2.6. Ethical Approval

Ethical approval for this study was obtained from the Committee on Human Research Publications and Ethics (CHRPE) of KNUST (Ref: CHRPE/AP/080/21). All participants provided informed consent prior to their inclusion in this study.

## 3. Results

### 3.1. Sociodemographic Characteristics of Participants

Table 1 shows that of the 171 participants involved in the study, 81 (22 males and 59 females) were from the rural community while 90 (59 males and 31 females) were from the urban community. In the rural community, the majority (53.1%) of the participants relied on tap water as their source of drinking water compared to 13.3% in the urban community.

### 3.2. Hydration Status of Participants

The overall prevalence of dehydration in the present study was 80% in the rural community while that of the urban community was 92.6% based on urinalysis with a strip. Specifically, 58.9% of urban dwellers and 85.2% of rural dwellers were minimally dehydrated, while 21.1% and 7.4% were significantly dehydrated in urban versus rural communities, respectively (Table 2). The findings thus found a lesser proportion of the participants from urban (20%) and rural (7.4%) communities to be well hydrated (Table 2). The findings also showed a significant difference (*p* = 0.0001) in the population density under the various hydration statuses, as the majority of the participants were dehydrated (minimal and significant dehydration).

There was a significant difference (*p* = 0.0001) in the hydration status of the participants from the urban and rural communities with more people being minimally dehydrated in the rural communities compared to the urban communities; however, the significantly dehydrated cases were higher in the urban communities than the rural communities. Using an optical refractometer, 68.9% of participants in the urban community were found to be minimally dehydrated, 20% as significantly dehydrated, and 11.1% as well hydrated. In comparison, 90.1% of participants in the rural community were minimally dehydrated, and 9.9% were significantly dehydrated (Table 2).

The urine specific gravity as index was determined, and the outcome indicated the results obtained from both equipment are comparable and almost the same (*p* = 0.8871).

The sensitivity of the urinalysis strip using the refractometer as the gold standard was 0.84 implying the test was able to detect 84% of the truly dehydrated participants as dehydrated. In comparison, 16% of the cases were undetected (specificity = 0.16) as false negatives (Table 3).

### 3.3. Physicochemical Quality of Water Samples Collected—pH

The pH of water samples collected from the urban community was between 5.99 and 7.12 while that from the rural community was between 5.51 and 6.17 as presented in Table 4. The pH results of the water samples infer that most of the samples are slightly acidic.

### 3.4. Microbial Quality of Water

#### 3.4.1. Total Aerobic Count

**Figure 1:**
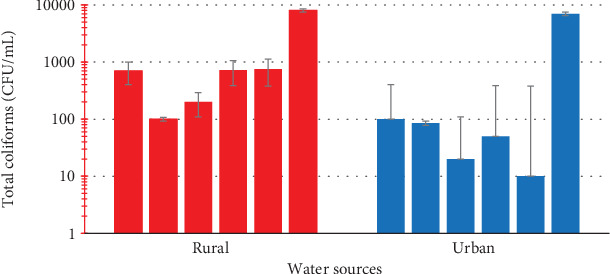
Total aerobic bacterial count of water samples from urban and rural communities.

Figure 1 shows that the water sampled from the urban communities recorded total aerobic bacteria count between 7.0 × 10^0^ ± 1.15 and 4.27 × 10^3^ ± 2.08 cfu/mL compared to the samples from the rural communities which had aerobic bacteria contamination levels between 9.2 × 10^1^ ± 7.64 and 5.93 × 10^3^ ± 5.51 cfu/mL. Considering the unit samples, 40% of the rural water samples recorded aerobic bacteria levels that exceeded the safe limit of 5.0 × 10^2^ cfu/mL [27], while 20% from the urban community failed the quality test. This notwithstanding, the mean aerobic bacterial water loads from the rural and urban communities did not differ significantly (*p* = 0.0649).

#### 3.4.2. Coliform and Pathogenic Bacteria Contamination of Water

**Figure 2:**
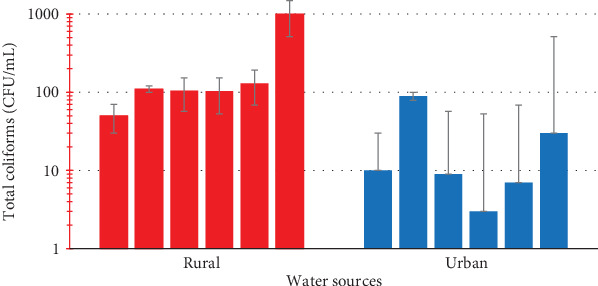
Coliform contamination levels of water from urban and rural communities.

The water from the urban communities were contaminated by coliforms with recorded loads between 2.0 × 10^0^ and 5.8 × 10^1^ ± 7.09 cfu/mL as well some significantly higher coliform levels between 2.8 × 10^1^ ± 2.89 and 7.96 × 10^2^ ± 5.86 cfu/mL from the water samples from rural communities (*p* = 0.0043) (Figure 2). Further investigation indicated no fecal coliforms were detected in all water samples from both urban and rural communities and characteristic of this was the absence of *Escherichia coli* which is a known indicator pathogen for fecal coliforms in all water samples (Table 5).

The results again established the absence of *Salmonella* in all water samples from both communities, which goes to further affirm the dominant Gram-negative bacteria in the water samples to be the Gram-negative cocci.

The findings again highlight the absence of *Staphylococcus aureus* in the water samples as well as the absence of other phenotypically distinct *Staphylococcus* species including *Staphylococcus epidermidis* and *Staphylococcus saprophyticus* on MSA. However, the HPC (heterotrophic plate count)/TAC (total aerobic count) plates indicated the presence of Gram-positive cocci which is suggestive of *Micrococci* species and *Diplococcus* in the water samples.

Gram differential staining of the colonies indicated the absence of the typical Gram-negative rods characteristic of coliforms and *Enterobacteriaceae* but rather small circular tetrad/cluster cells indicative of *Moraxella* species (Table 5). In the urban community, the majority (86.7%) of the participants did not rely on tap water which was readily available as a drinking source. However, most rural dwellers (53.1%) relied on tap water for drinking. Most urban dwellers did not trust tap water to be safe (78.9%), but most rural dwellers thought it was safe (61.7%), as presented in Table 5.

## 4. Discussion

In the Kumasi Metropolis, it was observed that majority of the participants, 92.6% of urban dwellers and 80% of rural dwellers, were dehydrated. This observation is concerning as dehydration has been proven to cause headaches, light headedness, increased blood pressure, kidney dysfunction, and cognitive defects. Majority of the participants purchased industrially purified water for drinking with others relying on tap water and borehole water. The buying of water was stated by some participants as a contributor to them not staying adequately hydrated. Though pathogenic bacteria such as *E. coli*, *Salmonella*, and *Staphylococcus aureus* were absent, the high mean total aerobic count and presence of coliforms in all samples exceeding safe limits are indicative of potential contamination and bacterial regrowth, thus requiring water to be retreated before use for domestic purposes such as cooking and drinking. This study contributes to literature on water safety within the region and is one of the premier studies assessing hydration status in the Kumasi Metropolis of Ghana.

The combined number of female participants from the present study was 90 which formed the majority of participants. This observation is similar to that observed by Appiah-Effah et al. [17] where females constituted the majority of their study participants. In the rural community, the majority (53.1%) of the participants relied on tap water as their source of drinking water compared to the 13.3% in the urban community. Other sources of water for urban dwellers included purified sachet and bottled water while rural dwellers largely relied on sachet water, bottled water, rainwater, and borehole. These other water sources are similar to sources observed in another study in the Kumasi suburb [17].

Based on the urinalysis assessment of hydration, the majority of the participants from both the rural (58.9%) and urban (85.2%) centers were minimally dehydrated. More rural dwellers (20.0%) than urban dwellers (7.4%) were well hydrated (*p* = 0.0001). Even though largely minimal, this observation of dehydration is concerning since hydration is necessary for optimal cognitive performance and mood regulation [28], especially for the urban population since they are students and largely rely on their cognition for academic excellence. Beyond cognitive performance, habitual dehydration has been found to increase the risk of cardiovascular diseases via its impairment of blood pressure and vascular function [29]. Though the evidence is inconsistent, dehydration has also been observed to affect other health outcomes including headaches and metabolic and digestive disorders [30].

Physicochemical analyses revealed that the pH of water from the urban community ranged from 5.99 to 7.12 while that of the rural community was 5.51 to 6.17 which are all within the Ghana Standards Authority and World Health Organization [31] recommended values for pH. The observed pH which indicates acidity is consistent with previous studies in Kumasi which have recorded acidic pH for groundwater [32] as well as potable water in households [17]. The acidity of groundwater previously reported might have accounted for the observed acidity in the present study since the tap water that the various companies supply is largely sourced from groundwater and streams. It is worth noting that the observed acidic pH range in the present study does not really pose a health risk since these are weak acids.

On microbial analyses, it was observed that the TAC of both urban and rural water sources exceeded the safe limit of 5.0 × 10^2^ cfu/mL by 20% and 40%, respectively. High TAC in water for domestic use can have significant health implications as it is a measure of aerobic micro-organisms including bacteria, fungi, and yeast [31]. It also suggests poor water quality and possible contamination from sewage, agricultural runoff, or industrial pollution [33]. Utilization of water contaminated with TAC could plausibly lead to gastrointestinal and skin infections. Further investigation of the TAC plates revealed the presence of Gram-positive cocci which is suggestive of *Micrococci* species and *Diplococcus* in the water samples. This observation is indicative of potential environmental contamination which could pose health risks to individuals using the water for domestic purposes such as drinking and cooking which raises public health concerns due to the threat and risk of communicable disease outbreak in the communities from utilizing unwholesome water for domestic activities.

Furthermore, water from both urban and rural communities were contaminated with coliforms loads of 2.0 × 10^0^–5.8 × 10^1^ ± 7.09 cfu/mL (urban) and 2.8 × 10^1^ ± 2.89–7.96 × 10^2^ ± 5.86 cfu/mL (rural), respectively. The rural contamination of coliforms was significantly higher than that in urban center (*p* = 0.0043). Total coliforms in themselves may not pose any health risk; however, it is a pointer to the presence of potentially harmful microbes such as *E*. *coli* which makes water unsafe for drinking [33, 34]. Further investigation indicated the absence of fecal coliforms in any of the samples. Even though *E*. *coli* was not detected, the presence of total coliforms is an indicator of the presence of other microbial contamination which could result in gastrointestinal illnesses. According to Public Health Ontario [34], the presence of total coliforms in water could indicate bacterial regrowth which requires further treatment before use to avoid health complications. In addition to gastrointestinal illnesses, chronic exposure to contaminated water can lead to persistent health problems including recurrent infections and nutritional deficiencies [31, 35, 34]. Thus, it is important for tap water to be treated before use to prevent any incidence of adverse health effects.

In the urban community, the majority of the participants (86.7%) did not rely on tap water which was readily available as a drinking water source. On the contrary, more rural dwellers (53.1%) relied on tap water for drinking. This is quite alarming as microbial analysis revealed that water from both sources was contaminated and thus required further treatment before use. However, we did not assess whether or not people who relied on tap water for drinking treated it or not before its use. On the perception of the safety of tap water, the majority of the urban participants (78.9%) did not think it was safe which is why they did not use it for drinking and cooking. On the other hand, 61.7% of rural dwellers thought it was safe. This disparity in perception could be attributed to urban dwellers having access to other sources of water and may be well informed through media or research and may possibly have had some bad experiences in the past using tap water for drinking or cooking. On the contrary, rural communities in Ghana often have limited access to pipe-borne water and might see it as safer to drink compared with ground-dug wells, boreholes, and surface water which they are mostly accustomed to.

## 5. Conclusion

Findings from the present study have revealed that the majority of the participants were not well hydrated. More rural dwellers relied on tap water for drinking than those in the urban community. Furthermore, tap water which remains a drinking option for some individuals was contaminated with nonpathogenic micro-organisms. Though no pathogenic micro-organisms were found, the presence of other coliforms is a cause for concern as it is indicative of bacterial regrowth.

### 5.1. Recommendation

There should be public education on the benefits of staying hydrated. Authorities should ensure frequent monitoring and evaluation of tap water since a good number of people rely on it for their source of livelihood.

### 5.2. Limitations

This study mainly studied the safety and use of tap water as a major source of hydration. The assessment of the microbial levels of all other water sources must be explored in future studies.

## Figures and Tables

**Table 1:** Sociodemographic characteristics of participants.

**Parameter**	**Rural community (** **n** = 81**)**	**Urban community (** **n** = 90**)**
*Age (years)*		
Mean (SD)	19.05 (10.79)	19 (1.04)
Range	10–61	17–22
*Gender*	*n* (%)	*n* (%)
Male	22 (27.2%)	59 (65.6%)
Female	59 (72.8%)	31 (34.4%)
*Tap water as source of drinking water*		
Yes	43 (53.1%)	12 (13.3%)
No	38 (46.9%)	78 (86.7%)
*Other sources of drinking water*		
Purified sachet only	29 (35.8%)	12 (13.3%)
Purified sachet and bottled water	35 (43.2%)	78 (86.7%)
Purified sachet, bottled water, and rainwater	2 (2.5%)	—
Purified sachet, bottled water, and borehole	14 (17.3%)	—
Purified sachet and borehole	1 (1.2%)	—
*How often do you drink water?*		
Sometimes	52 (64.2%)	32 (35.6%)
Very often	29 (35.8%)	58 (64.4%)

Abbreviation: SD, standard deviation.

**Table 2:** Hydration status and descriptive statistics of hydration status among urban and rural community participants.

**Hydration status/measure**	**Rural (** **N** = 81**)**	**Urban (** **N** = 90**)**	**p** ** value**
Total dehydration			
Urinalysis strip	80%	92.6%	
Refractometer	88.9%	100%	
Minimal dehydration			0.0001
Urinalysis strip	58.9%	85.2%	
Refractometer	68.9%	90.1%	
Significant dehydration			
Urinalysis strip	21.1%	7.4%	
Refractometer	20.0%	9.9%	
Well hydrated			0.0001
Urinalysis strip	20.0%	7.4%	
Refractometer	11.1%	0%	

**Type of settlement**	**N**	**Range**	**M** **e** **a** **n** ± **SD**
Urinalysis strip			
Rural	81	1.005–1.030	1.021 ± 0.023
Urban	90	1.010–1.025	1.018 ± 0.007
Refractometer			
Rural	81	1.003–1.030	1.017 ± 0.003
Urban	90	1.012–1.024	1.018 ± 0.006

**Table 3:** Association between refractometry and urinalysis strip assay in determining hydration status using Fisher's exact test.

**Variables**	**Test**	**p** ** value**	**Odds ratio**	**Sensitivity**	**Specificity**
Refractometer vs. urinalysis	Fisher's exact test	0.8871	1.067	0.8413	0.1676

**Table 4:** pH of water samples from urban and rural communities in Ashanti Region.

**Source**	**001**	**002**	**003**	**004**	**0005**	**006**	**p** ** value**
Urban	6.62 ± 0.01	6.87 ± 0.01	6.06 ± 0.01	6.64 ± 0.014	5.99 ± 0.02	7.12 ± 0.03	0.0001
Rural	6.17 ± 0.06	5.56 ± 0.02	5.54 ± 0.01	5.85 ± 0.02	5.51 ± 0.01	5.96 ± 0.25	0.0038
	*p* = 0.0042						

*Note:* 001–006: water samples from six randomly selected taps in both urban and rural communities.

**Table 5:** Presence of pathogenic bacteria, micromorphological characterization of bacteria, and participant's views on tap water as drinking water.

**Sample code**	**Community**	***E. coli*/100 mL**	***S. aureus*/mL**	***Salmonella*/100 mL**	**Media**	**Water sources**	**Arrangements**	**Reaction**	**Shape**	**Inference**	**Rely on tap water (** **N** ** , %)**	**Trust tap water is safe (** **N** ** , %)**
001	Urban	Not detected	0.00	Not detected	PCA	U001	Cluster	Positive	Coccus	Micrococci	Yes: 12 (13.3)	Yes: 19 (21.1)
002	Urban	Not detected	0.00	Not detected	PCA	U002	Cluster	Positive	Coccus	Micrococci	No: 78 (86.7)	No: 71 (78.9)
003	Urban	Not detected	0.00	Not detected	PCA	U003	Cluster	Negative	Coccus	Moraxella		
004	Urban	Not detected	0.00	Not detected	PCA	U004	Cluster	Negative	Coccus	Moraxella		
005	Urban	Not detected	0.00	Not detected	PCA	U005	Cluster	Negative	Coccus	Moraxella		
006	Urban	Not detected	0.00	Not detected	PCA	U006	Cluster	Positive	Coccus	Micrococci		
001	Rural	Not detected	0.00	Not detected	PCA	R001	Cluster	Negative	Coccus	Moraxella	Yes: 43 (53.1)	Yes: 50 (61.7)
002	Rural	Not detected	0.00	Not detected	PCA	R002	Cluster	Positive	Coccus	Micrococci	No: 38 (46.9)	No: 31 (38.3)
003	Rural	Not detected	0.00	Not detected	PCA	R003	Cluster	Negative	Coccus	Moraxella		
004	Rural	Not detected	0.00	Not detected	PCA	R004	Cluster	Negative	Coccus	Moraxella		
005	Rural	Not detected	0.00	Not detected	PCA	R005	Cluster	Negative	Coccus	Moraxella		
006	Rural	Not detected	0.00	Not detected	PCA	R006	Cluster	Positive	Coccus	Micrococci		

*Note:* U001–U006: water samples from urban communities, R001–R006: water samples from rural communities.

Abbreviations: MA, MacConkey agar; PCA, plate count agar.

## Data Availability

The data that support the findings of this study are available from the corresponding author upon reasonable request.

## Nomenclature

PCA: plate count agar
MA: MacConkey agar
MSA: mannitol salt agar
XLD: xylose lysine deoxycholate agar
CHRPE: Committee on Human Research Publications and Ethics
KNUST: Kwame Nkrumah University of Science and Technology
SPSS: Statistical Package for the Social Sciences
ANOVA: analysis of variance
